# Tuning of tetrathiafulvalene properties: versatile synthesis of *N*-arylated monopyrrolotetrathiafulvalenes via Ullmann-type coupling reactions

**DOI:** 10.3762/bjoc.11.96

**Published:** 2015-05-21

**Authors:** Vladimir A Azov, Diana Janott, Dirk Schlüter, Matthias Zeller

**Affiliations:** 1 Department of Chemistry, University of Bremen, Leobener Str. NW 2C, D-28359 Bremen, Germany; 2 One University Plaza, Department of Chemistry, Youngstown State University, Youngstown, OH 44555-3663, USA

**Keywords:** cyclic voltammetry, *N*-arylation, pyrrolotetrathiafulvalene, Ullmann-type coupling, X-ray crystallography

## Abstract

An Ullmann-type coupling reaction was employed for the preparation of several *N*-arylated monopyrrolotetrathiafulvalenes with variable substitution patterns. Spectroscopic and electrochemical properties of the coupling products strongly depend on the electronic nature of the aromatic substituents due to their direct conjugation with the tetrathiafulvalene chromophore. The crystal packing of the arylated monopyrrolotetrathiafulvalenes is primarily defined by networks of C–H···X weak hydrogen bonds and short S···S contacts involving the tetrathiafulvalene moieties.

## Introduction

**Figure 1:**
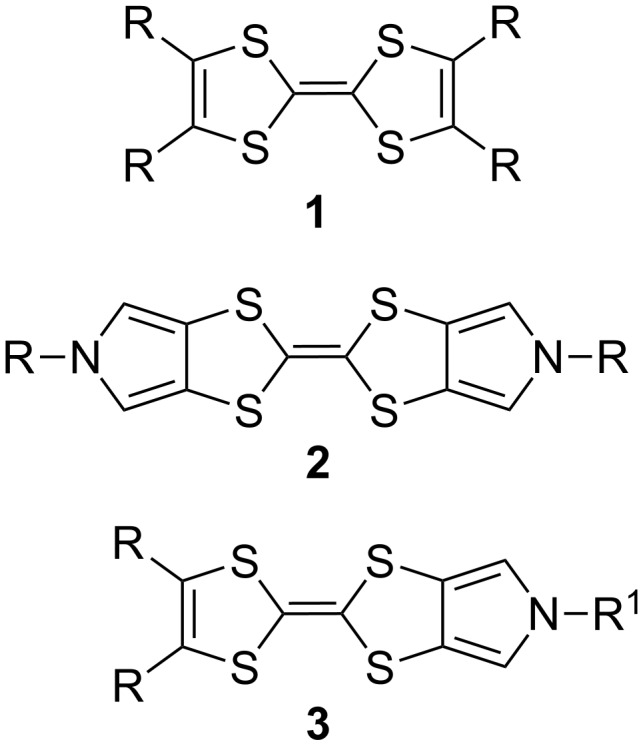
Molecular structures of tetrathiafulvalenes **1**, bis-pyrrolotetrathiafulvalenes **2** and monopyrrolotetrathiafulvalenes **3**.

For the last four decades tetrathiafulvalenes [1–2] (Figure 1) **1** have been the subject of extensive studies due to their outstanding electron-donating properties and ability to induce reversible electrochemically-induced switching processes in molecular and supramolecular systems [3–4]. Availability of selective synthetic methods [5–6] gave access to differently substituted tetrathiafulvalene (TTF) moieties which allowed tuning of oxidation potential, donating ability, as well as other physical and chemical properties. The regioselective functionalization of TTF, however, remains problematic due to the presence of four identical attachment sites. Incorporation of the TTF moiety in macrocycles usually leads to poorly separable mixtures of *cis/trans* isomers [7–9]. Even if separation is possible, TTFs are prone to *cis/trans* isomerization, which can be induced by light [10] or traces of acid [11]. These problems are aggravated by the fact that each reversible oxidation–reduction cycle of the TTF moiety always leads to formation of *cis/trans* isomer mixtures.

**Scheme 1:**
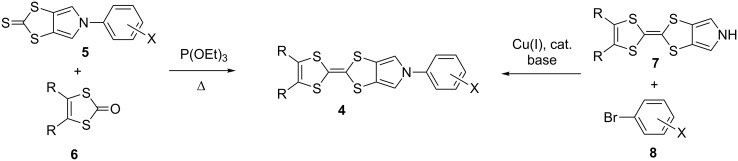
The two synthetic approaches used for the preparation of arylated monopyrrolotetrathiafulvalenes **1**.

Bis-pyrrolotetrathiafulvalenes **2** and monopyrrolotetrathiafulvalenes (MPTTFs) **3** represent a significant modification of the TTF backbone featuring a more extended electron-rich π-system with only two or three easily accessible attachment points for external substituents, respectively [12–13]. The asymmetric nature of MPTTFs **3** opens the possibility for the introduction of different R- and R^1^-groups on the two sides of the TTF moiety. If R^1^ = alkyl (or a similar group with an sp^3^-hybridized carbon atom), such substituents can be readily attached to the pre-formed MPTTF moiety using a variety of common nucleophilic substitution reactions. In the case of R^1^ = aryl, two possible approaches for the preparation of *N*-arylated MPTTF derivatives **4** have been reported (Scheme 1). In the first procedure [14–16], the aryl substituent is incorporated during the initial synthetic steps to form a *N*-aryl-1,3-dithiolo[4,5-*c*]pyrrole-2-thione **5**, which is then coupled to 1,3-dithiole-2-thione **6** in hot triethyl or trimethyl phosphite. Using the second approach [17–19], the aryl group is attached to the MPTTF moiety using a direct copper-mediated Ullmann-type *N*-arylation reaction [20–21]; this method was also used for the preparation of arylated bis-pyrrolotetrathiafulvalenes **2**. Although being reported in the literature, it has so far not found wide spread use and was employed only with a narrow scope of aromatic derivatives with electron-donating substituents and alkylthio-substituted (R = SAlkyl) MPTTFS.

Being interested in the preparation of calix[4]arene receptors with MPTTF moieties directly attached to an aromatic calixarene backbone, we have chosen the copper-catalysed *N*-arylation reaction as a method for coupling of aromatic and MPTTF moieties with each other and successfully employed it for the preparation of two bis-MPTTF-calixarene conjugates, as well as two model low molecular weight aromatic derivatives **4a** and **4c** [22]. To explore the scope of the reaction, we decided to conduct a deeper investigation varying reaction conditions and testing different substituents on the aromatic as well as the MPTTF components of the reaction. It led to the preparation of a family of MPTTF aromatic derivatives, whose properties and crystal structures are discussed below.

## Results and Discussion

### Synthesis

**Scheme 2:**
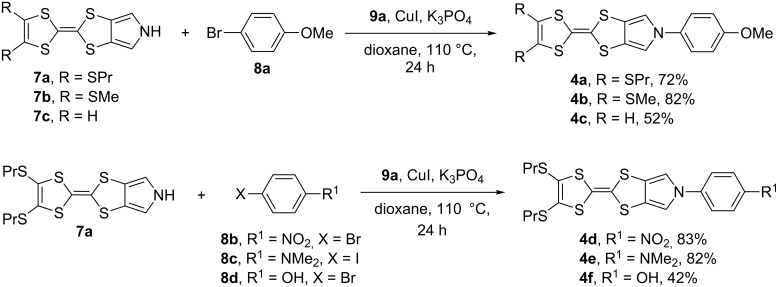
Synthesis of arylated monopyrrolotetrathiafulvalenes **4a–f**.

**Figure 2:**
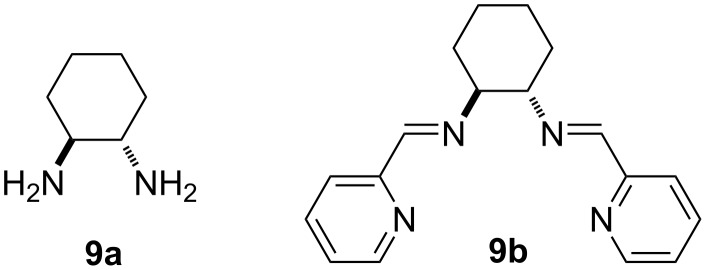
Copper(I) ligands **9a** and **9b**.

Our initial synthetic efforts were focused on optimizing reaction conditions using the PrS-MPTTF derivative **7a** [23] and bromoanisole **8a** as starting materials (Scheme 2), as well as comparing two possible copper(I) ligands (Figure 2): *trans*-diaminocyclohexane (DACH) **9a**, which was employed before in *N*-arylations with pyrrolo-TTFs [17–19], and its Schiff base derivative **9b**, which was reported to be one of the most effective ligands in similar *N*-arylation reactions with other substrates [20–21].

Initial experiments gave evidence of much lower efficiency of ligand **9b** in comparison to **9a** in terms of conversion and presence of undesired side-products. Reaction utilizing *trans*-diaminocyclohexane **9a**also allowed for fast optimization and reproducibility, which prompted us to focus our attention on the CuI/**9a** catalysis system. Although copper(I) is supposed to play a catalytic role [20–21], smaller CuI loadings led to diminished reaction yields, supposedly due to lower solubility/activity of the CuBr formed in the coupling reaction. Taking into account the affordability of CuI, its excess cannot be considered a disadvantage of the method. Additionally, use of the inexpensive Cu(I) catalyst allows to avoid Buchwald–Hartwig amination [24–25], which employs more expensive Pd-based catalysts for a similar type of C–N coupling reactions.

In a typical procedure, 1 equiv of MPTTF **7a**, **7b** [12], or **7c** [26], 1.5–1.6 equiv of a brominated aromatic derivative, 0.5 equiv of CuI/**9a** and 3–4 equiv of K_3_PO_4_ were heated at 110–115 °C overnight in absolute dioxane in a Schlenk tube. The reaction yields amounted to 70–80% for stable MPTTF derivatives **4a,b,d,e**, but were lower for **4c** due to sensitivity of the starting material **7b**, as well as for **4f** due to its tendency towards oxidation.

Thus, the *N*-arylation reaction can be readily employed with electron-rich as well as electron-deficient aromatic derivatives, as well as with thioalkyl-substituted and non-substituted MPTTFs. The successful reaction with bromophenol **8d** to form the adduct **4f** also confirmed the possibility of the reaction with hydroxy-substituted aryl derivatives, paving the way for application of this method with non-protected calix[4]arene derivatives [27].

**Figure 3:**
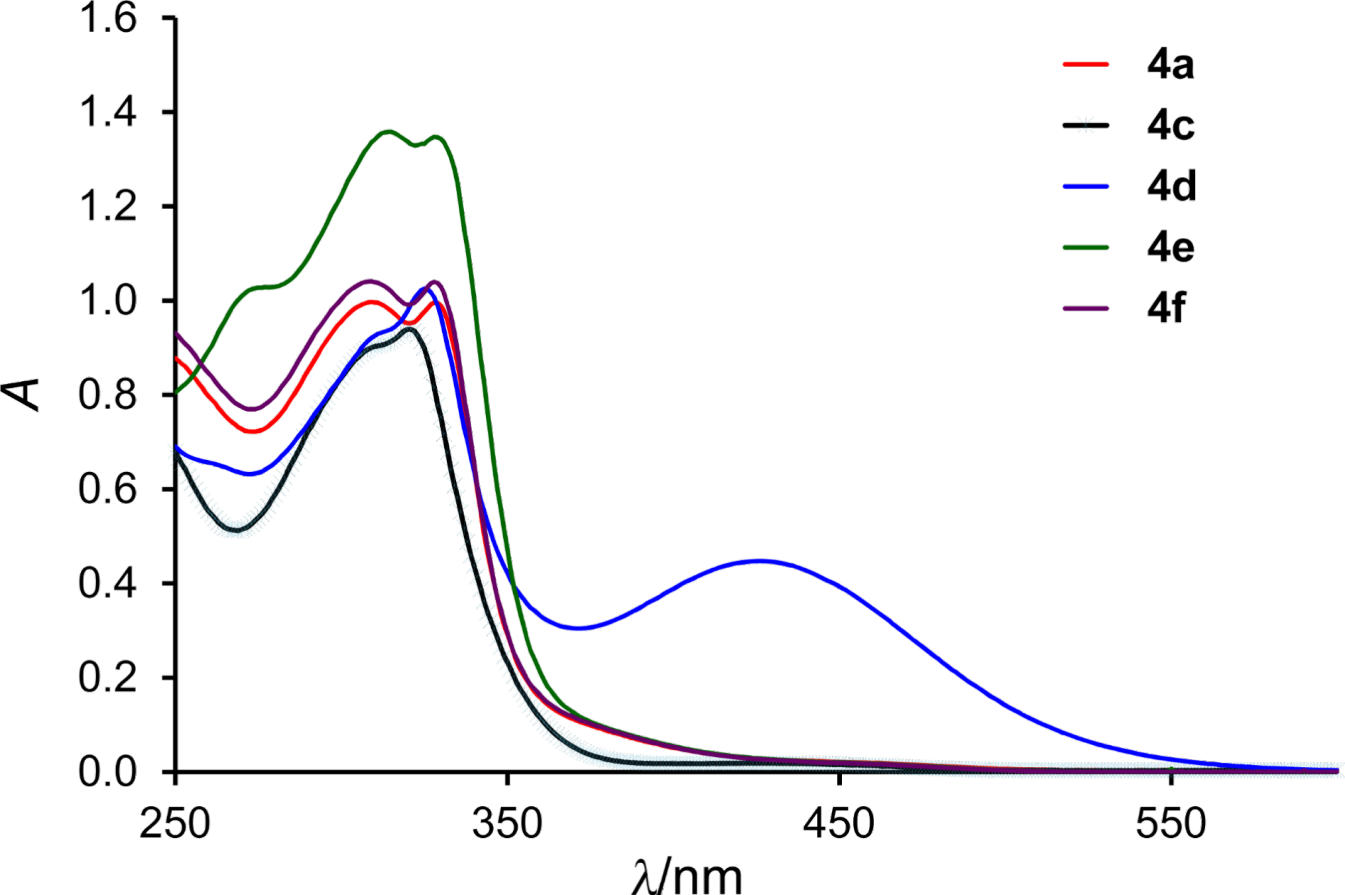
UV–vis spectra of compounds **4a,c–f** (CH_2_Cl_2_, *c* = 4 × 10^−5^ M).

Compounds **4a–c,e,f** (Figure 3) display UV–vis spectra typical for TTF derivatives with absorption maxima at λ_max_ of ca. 310–330 nm as well as a long tail with very low absorption spanning to ca. 500 nm and rendering the yellow colour to the compounds. Thioalkyl TTF derivatives **4a,b,e,f** also show a sloping shoulder at ca. 390 nm, which is missing in the non-substituted **4c**. In contrast, the spectrum of nitro-derivative **4d** displays an additional strong absorption band centred at ca. 425 nm, arising most likely due to charge transfer from the electron-rich MPTTF moiety to the electron-deficient aromatic substituent. This absorption manifests itself in the dark red colour of **4d**, both in solid state as well as in solution.

MPTTF derivatives are readily soluble in non-polar organic solvents, such as CH_2_Cl_2_, toluene or acetone (with the exception of poorly soluble **4c**), giving solutions that are stable at room temperature in air.

### Electrochemistry

**Figure 4:**
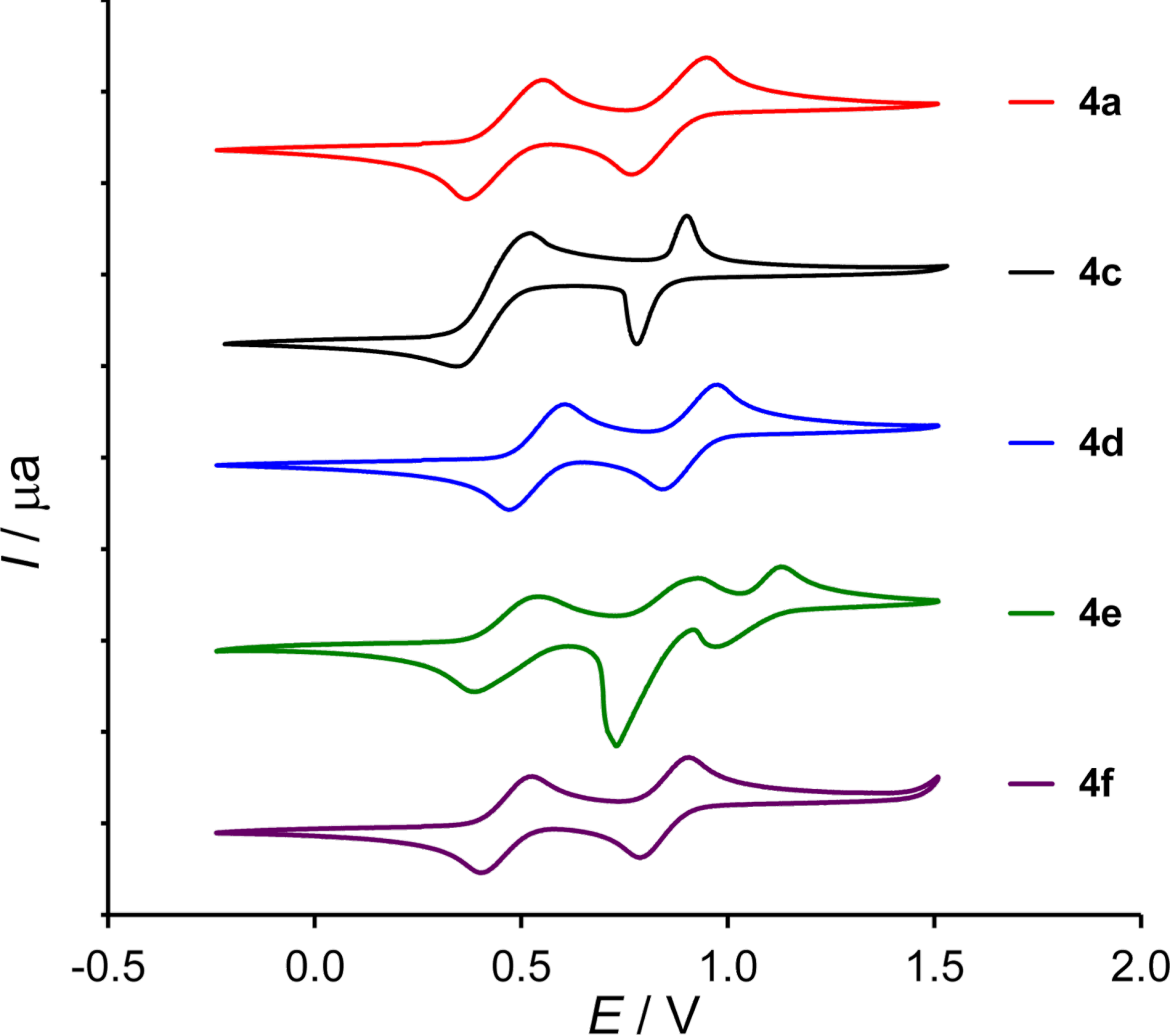
Cyclic voltammograms of compounds **4a,c** [22] and **4d–f** (plotted vs SCE; CH_2_Cl_2_/0.1 M Bu_4_NClO_4_).

Solution oxidation potentials of aromatic MPTTF conjugates were determined using cyclic voltammetry (CV) in CH_2_Cl_2_/Bu_4_NClO_4_ solution and are summarized in Table 1. The CVs of all compounds displayed two reversible oxidation waves on the cathodic scan (Figure 4) characteristic to TTFs [1], the first one leading to the radical cation and the second to the dication. Non-substituted derivative **4c** shows a lower first oxidation potential than its alkylS-substituted counterpart **4a,b**, as expected due to the electron-withdrawing effect of the two thioalkyl groups [28–29]. The strong electron-withdrawing effect of the 4-nitrophenyl group in **4d** manifests itself in an increased oxidation potential with a shift of ca. 0.08 V for both oxidation waves. Aromatic electron-donating groups as in **4e** and **4f** do barely influence the potential of the two oxidation waves. Instead, they induce an additional oxidation wave at higher potentials of 1.06 V and 1.77 V, in **4e** and **4f** (see Figure S7, Supporting Information File 1), respectively. For the phenol derivative **4f**, this oxidation is irreversible.

**Table 1:** Electrochemical data.^a^

Compound	*E*_1/2_^ox1^ (V)	*E*_1/2_^ox2^ (V)	*E*_1/2_^ox3^ (V)

**4a** [22]	0.47	0.84	–
**4b**	0.48	0.83	–
**4c** [22]	0.40	0.84	–
**4d**	0.55	0.92	–
**4e**	0.46	0.84	1.06
**4f**	0.47	0.86	1.76 (irrev.)

^a^Data were obtained using a one-compartment cell in CH_2_Cl_2_/0.1 M Bu_4_NClO_4_, Pt as the working and counter electrodes and a non-aqueous Ag/Ag^+^ reference electrode; scan rate 100 mV/s. Values given at room temperature vs SCE; the Fc/Fc^+^ couple (0.480 V vs SCE) was used as an internal reference [30].

### Crystal structures

Compounds **4a,b,d,e** afforded high quality crystals that could be analysed using X-ray crystallography, allowing to unambiguously confirm the identity of the compounds and analyse their structural properties and arrangement in the solid state. Bond lengths and angles in all structures may be considered normal. With the exception of alkylS-substituents, the molecular frameworks of **4a,b,d,e** display relatively low deviations from planarity. Angles between the least-square planes, defined by the heavy atoms of the aromatic ring and neighbouring pyrrole ring do not exceed 17.3° (in **4e**, see Table 2), ensuring good conjugation between the MPTTF and aromatic moieties. Boat-type deviations of the TTF groups (folding along the S–S vectors in the five-membered rings) are minor for electron-deficient **4d** lying below 5°, whereas they are much larger in electron-rich derivatives, where they reach 20.45° in **4a**, 13.58° in **4b** and 11.23° in **4e**. This observation corresponds well with previously reported data: the electron-deficient *N*-Ts derivative has an almost planar arrangement of the TTF moiety, whereas *N*-alkyl derivatives show significant deviation from planarity [12].

**Table 2:** Angles (°) between the planes of the aromatic ring and neighbouring pyrrole ring in **4a,b,d,e**.

Compound	Molecule1	Molecule 2

**4a**	3.41	3.42
**4b**	3.69	12.20
**4d**	1.36	10.68
**4e**	13.72	17.28

Interestingly, the crystal structures of all four compounds feature similar packing arrangements with two crystallographically distinct molecules (*Z*’ = 2) with quasi-parallel tilted edge-to face arrangements. In the crystal packing, molecules are interconnected by multiple non-classical weak intermolecular hydrogen bonds [31] and C–H···π and S···S interactions, the latter being common in the crystals of sulfur-rich compounds such as TTFs [32]. Most of the close contacts involve the central parts of neighbouring molecules, thus connecting them with each other and leading to formation of supramolecular layers. Parallel layers are only loosely bound to each other [33]. This layered arrangement manifests itself in the crystal morphology: all Ar-MPTTF derivatives crystallize in the form of thin platelets, with the plane of the parallel molecular layers coinciding with the direction normal to the largest crystal face of the platelets. This observation can be rationalized by assuming fast crystal growth within each supramolecular layer, assisted by the presence of directed weak hydrogen bonds as well as C–H···π and S···S interactions. Addition of new parallel layers, connected to the previous layers via dispersive interactions of the van der Waals type, can be assumed to be a much slower process, leading to plate like crystals that mimic the layered makeup at the molecular level. Crystals of **4d**, for example, form plates with an aspect ratio of 10 and above. Such layered arrangements make these derivatives possible candidates for organic electronics [34] and may serve as a motivation for evaluation of their electronic properties in the solid state.

**Figure 5:**
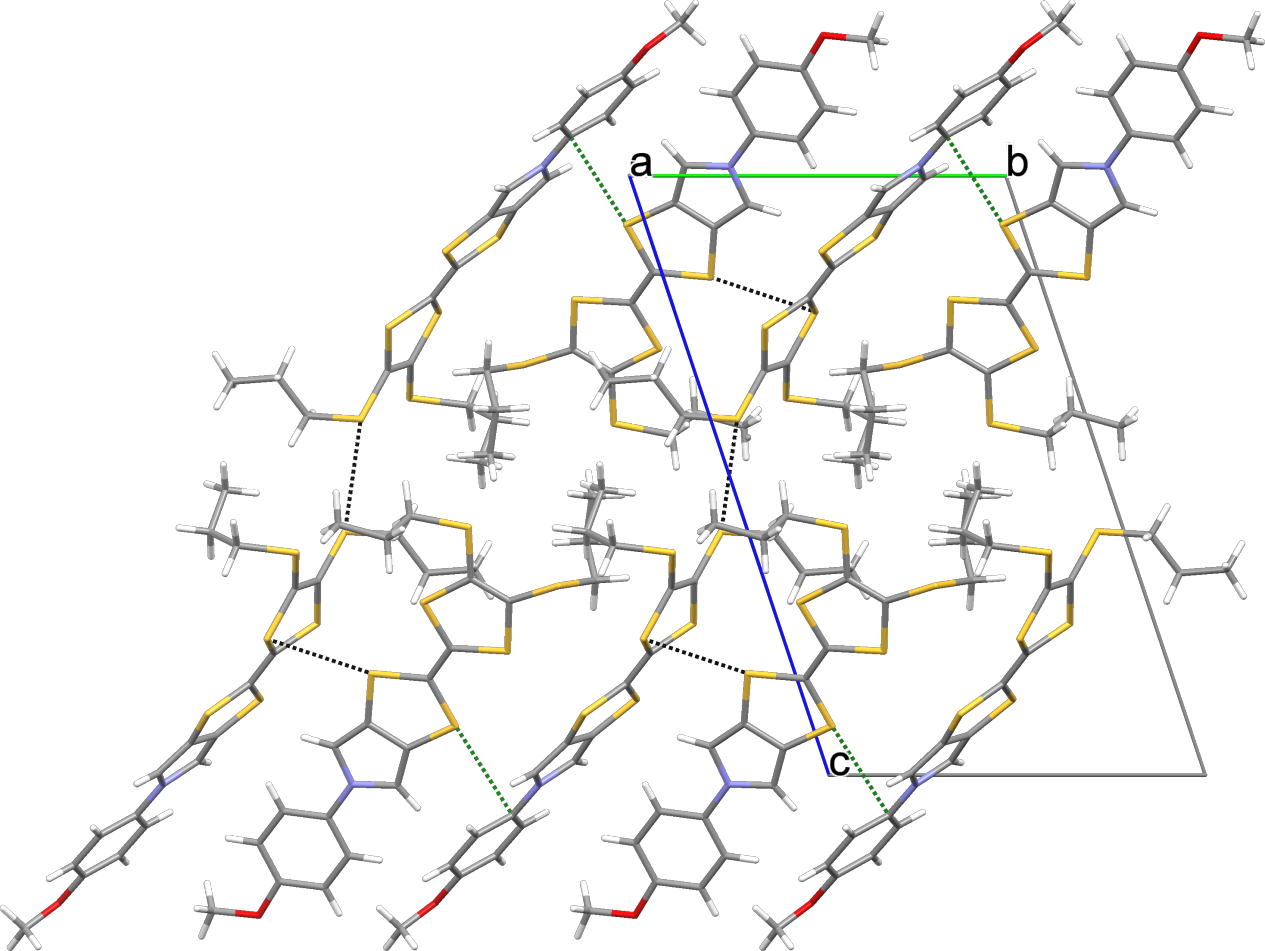
Crystal packing of **4a** viewed along the *a* axis and showing one layer of molecules. Short S···S contacts are shown as black and S···C contacts as green dashed lines. Only the major orientation of the disordered propyl chain is shown.

**Figure 6:**
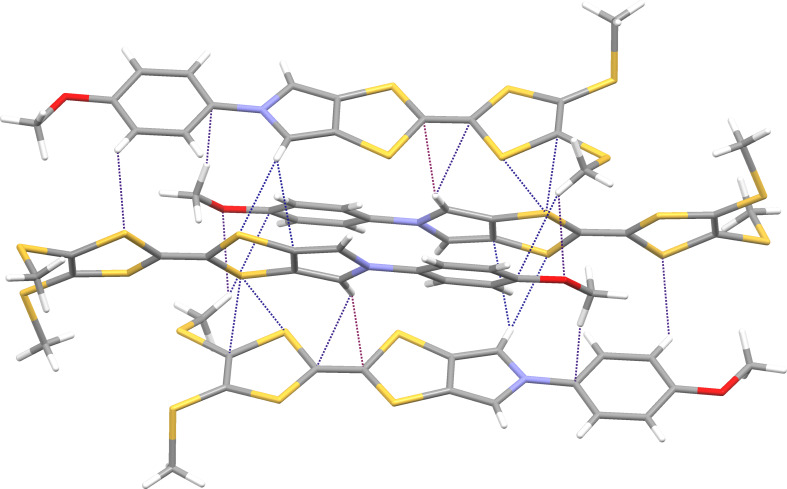
Crystal packing of **4b** showing a group of four molecules interconnected by multiple weak hydrogen bonds, C–H···π, and S···S close contacts.

**Figure 7:**
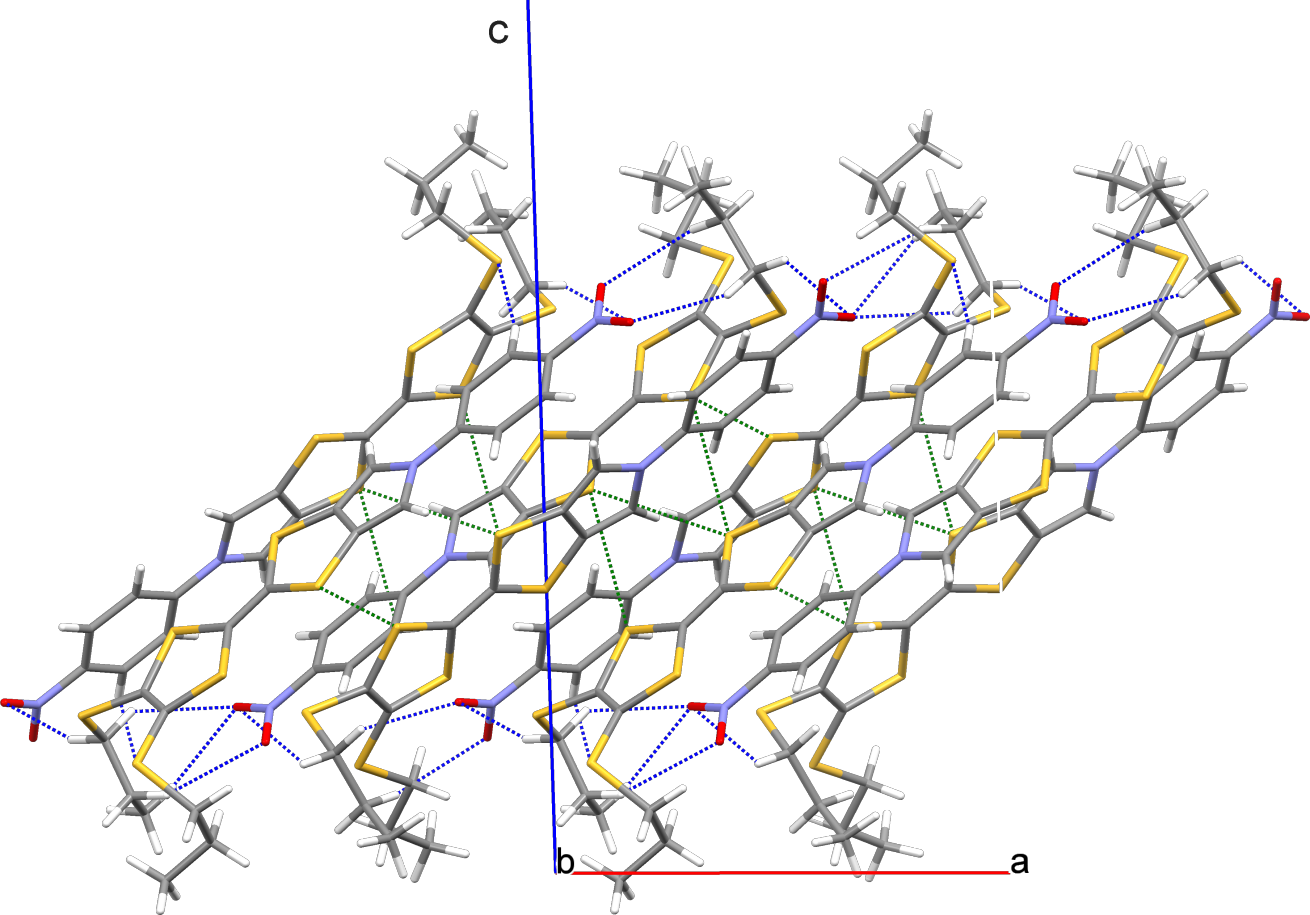
Crystal packing of **4d** viewed along the *b* axis. Molecules of **4d** form layers parallel to the (001) plane being interconnected with each other by means of short S···S contacts (green) and C–H···O_2_N weak hydrogen bonds (blue).

**Figure 8:**
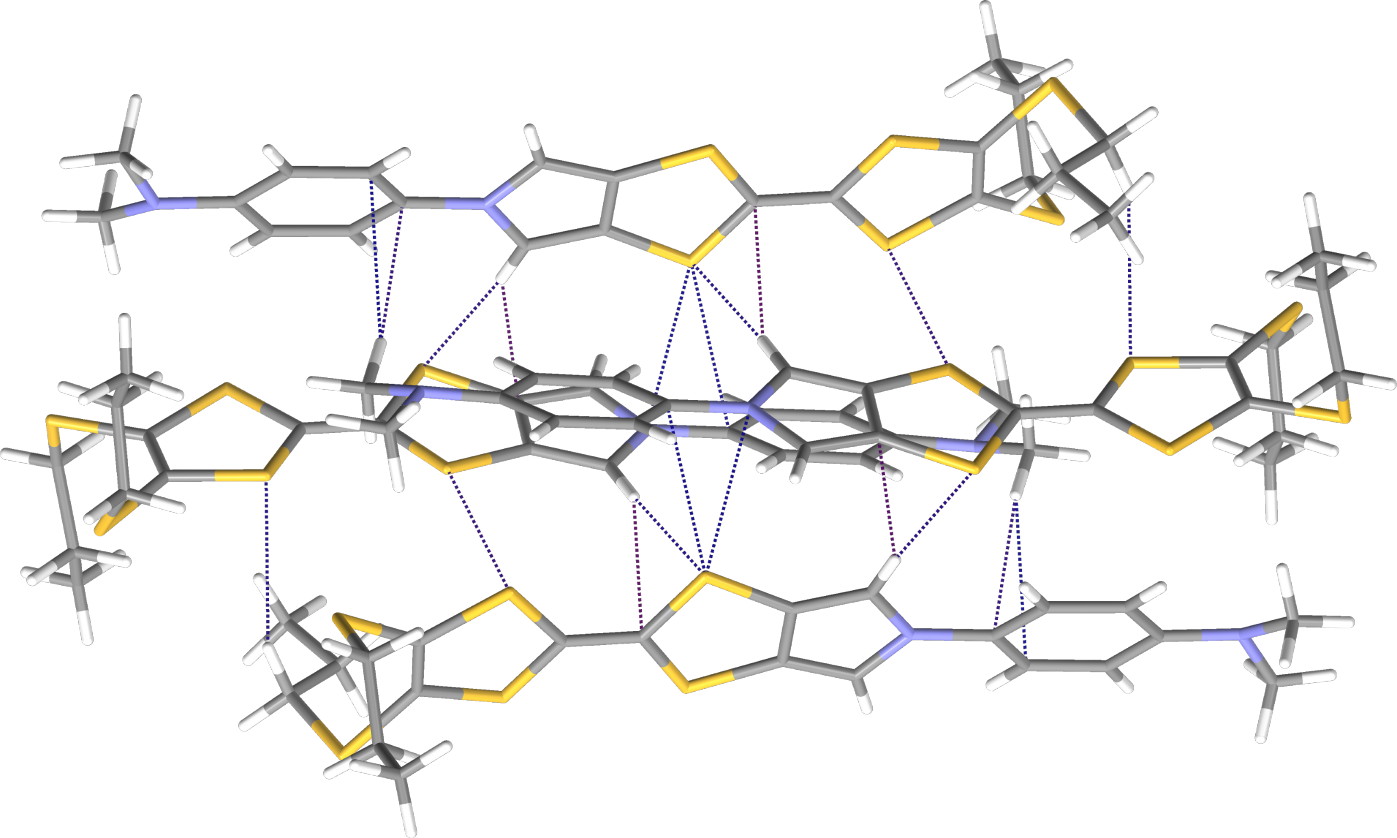
Crystal packing of **4e** showing a group of four molecules interconnected by multiple weak hydrogen bonds, C–H···π, and S···S close contacts.

Figures 5–8 display some aspects of molecular packing of MPTTF derivatives **4a,b,d,e**.

## Conclusion

In summary, several arylated monopyrrolotetrathiafulvalene derivatives have been conveniently prepared using a copper-mediated Ullmann-type *N*-arylation reaction. The reaction was shown to tolerate substituents of different nature on the aromatic group, as well as can be employed with substituted and non-substituted MPTTFs, opening the way for its application for the synthesis of more complex molecular systems, such as conjugates with non-protected calixarenes. New aromatic-MPTTF conjugates were characterized using different analytical methods and X-ray crystallography.

## Experimental

**Copper-catalyzed *N*-arylation reaction of monopyrrolo-tetrathiafulvalenes, general procedure.** The reaction was performed in a similar manner as described before [22]. A heavy walled Schlenk tube with a wide bore Teflon screw stopcock was charged with MPTTF derivatives **7a**, **7b** or **7c**, CuI, K_3_PO_4_, (±)-*trans*-1,2-diaminocyclohexane and an aromatic bromide, then absolute dioxane was added via syringe. The reaction mixture was degassed by three freeze-pump-thaw cycles, the vessel was filled with nitrogen, tightly sealed and stirred at 110–115 °C. The reaction was complete in 18–24 h (TLC control). The solvent was removed under reduced pressure directly from the Schlenk tube, the residue was dissolved in CH_2_Cl_2_, filtered through a plug of celite, and evaporated to dryness. The crude products were triturated with *n*-hexane to remove the unreacted aromatic starting material and then purified by flash chromatography on silica gel to afford pure *N*-arylated MPTTFs.

Preparation and characterization of 2-[4,5-bis(propylthio)-1,3-dithiol-2-ylidene]-5-(4-methoxyphenyl)-5*H*-1,3-dithiolo[4,5-*c*]pyrrole (**4a**) and 2-[1,3-dithiol-2-ylidene]-5-(4-methoxyphenyl)-5*H*-1,3-dithiolo[4,5-*c*]pyrrole (**4c**) was described before [22], also including the X-ray data of **4a** (see Supporting Information File 1 and Supporting Information File 2). For **4a**, corrected oxidation potentials are reported in Table 1.

**2-[4,5-Bis(methylthio)-1,3-dithiol-2-ylidene]-5-(4-methoxyphenyl)-5*H*-1,3-dithiolo[4,5-*c*]pyrrole (4b)**. Prepared from **7b** (0.040 g, 0.119 mmol), CuI (0.023 g, 0.119 mmol), K_3_PO_4_ (0.020 g, 0.954 mmol), *trans*-diaminocyclohexane (22 µL, 0.179 mmol) and 4-bromoanisole (**8a**, 0.033 g, 0.179 mmol) in 3 mL of dry dioxane. The product was purified by flash chromatography (CH_2_Cl_2_/cyclohexane, 1:2) to afford bright yellow crystals. X-ray quality crystals were grown by slow evaporation of CDCl_3_ solution. Yield: 43.2 mg (0.098 mmol, 82%). Mp 198–200 °C; *R*_f_ = 0.32 (CH_2_Cl_2_/cyclohexane, 1:2); ^1^H NMR (360 MHz, CDCl_3_) δ 7.25–7.20 (m, 2H), 6.95–6.91 (m, 2H), 6.79 (s, 2H), 3.83 (s, 3H), 2.43 (s, 6H); ^13^C NMR (90 MHz, CDCl_3_) δ 158.0, 134.0, 127.1, 121.9, 121.2, 120.2, 114.7, 111.4, 111.1, 55.6, 19.2; UV–vis (CH_2_Cl_2_) λ_max_ (ε): 309 nm (25600 L∙mol^−1^∙cm^−1^), 329 (24500); MS (EI) *m*/*z* (%): 441 (100) [M]^+•^, 426 (10) [M − Me]^+^; HRMS (EI) *m*/*z*: [M]^+•^ calcd for C_17_H_15_NOS_6_^+•^, 440.94780; found, 440.94675; CV (vs SCE, CH_2_Cl_2_): *E*_1/2_^ox1^ = 0.48 V, *E*_1/2_^ox2^ = 0.83 V.

**2-[4,5-Bis(propylthio)-1,3-dithiol-2-ylidene]-5-(4-nitrophenyl)-5*H*-1,3-dithiolo[4,5-*c*]pyrrole (4d).** Prepared from **7a** (0.055 g, 0.140 mmol), CuI (0.014 g, 0.070 mmol), K_3_PO_4_ (0.089 g, 0.42 mmol), *trans*-diaminocyclohexane (7.5 µL, 0.062 mmol) and 4-bromonitrobenzene (**8b**, 0.045 g, 0.224 mmol) in 2 mL of dry dioxane. The product was purified by flash chromatography (CH_2_Cl_2_) to afford deep red crystals. X-ray quality crystals were grown by slow evaporation of CDCl_3_/heptane solution. Yield: 59.7 mg (0.116 mmol, 83%). Mp 240–244 °C; *R*_f_ = 0.72 (CH_2_Cl_2_); ^1^H NMR (360 MHz, CDCl_3_) δ 8.33–8.29 (m, 2H), 7.44–7.40 (m, 2H), 6.99 (s, 2H), 2.81 (t, ^3^*J* = 7.2 Hz, 4H), 1.68 (sext, ^3^*J* = 7.2 Hz, 4H), 1.02 (t, ^3^*J* = 7.2 Hz, 6H); ^13^C NMR (90 MHz, CDCl_3_) δ 144.7, 144.2, 127.5, 125.8, 125.3, 118.6, 117.3, 113.7, 110.1, 38.2, 23.1, 13.2; UV–vis (CH_2_Cl_2_) λ_max_ (ε): 325 nm (25600 L∙mol^−1^∙cm^−1^), 426 (11200); MS (EI) *m*/*z* (%): 512 (100) [M]^+•^, 469 (5) [M − Pr]^+∙^, 436 (20) [M − HSPr]^+•^; HRMS (EI) *m*/*z*: [M]^+•^ calcd for C_20_H_20_N_2_O_2_S_6_^+•^, 511.98435; found, 511.98296; CV (vs SCE, CH_2_Cl_2_): *E*_1/2_^ox1^ = 0.55 V, *E*_1/2_^ox2^ = 0.92 V.

**2-[4,5-Bis(propylthio)-1,3-dithiol-2-ylidene]-5-(4-dimethylaminophenyl)-5*H*-1,3-dithiolo[4,5-*c*]pyrrole (4e).** Prepared from **7a** (0.050 g, 0.128 mmol), CuI (0.012 g, 0.063 mmol), K_3_PO_4_ (0.082 g, 0.386 mmol), *trans*-diaminocyclohexane (7.5 µL, 0.062 mmol) and 4-iodo*-N,N*-dimethylaniline (**8c**, 0.050 g, 0.202 mmol) in 2 mL of dry dioxane. The product was purified by flash chromatography (CH_2_Cl_2_/cyclohexane, 1:1) to afford light orange crystals. X-ray quality crystals were grown by slow evaporation of CDCl_3_/heptane solution. Yield: 53.8 mg (0.105 mmol, 82%); Mp 196–199 °C; *R*_f_ = 0.5 (CH_2_Cl_2_/cyclohexane, 1:1); ^1^H NMR (360 MHz, CD_2_Cl_2_) δ 7.22–7.17 (m, 2H), 6.81 (s, 2H), 6.76–6.71 (m, 2H), 2.96 (s, 6H), 2.81 (t, ^3^*J* = 7.2 Hz, 4H), 1.66 (sext, ^3^*J* = 7.2 Hz, 4H), 1.01 (t, ^3^*J* = 7.2 Hz, 6H); ^13^C NMR (90 MHz, CD_2_Cl_2_) δ 149.6, 130.7, 127.9, 122.0, 120.6, 119.8, 113.1, 111.9, 111.1, 40.8, 38.5, 23.5, 13.2; UV–vis (CH_2_Cl_2_) λ_max_ (ε): 314 nm (34000 L∙mol^−1^∙cm^−1^), 330 (33600); MS (EI) *m*/*z* (%): 510 (100) [M]^+•^, 434 (20) [M − HSPr]^+•^; HRMS (EI) *m*/*z*: [M]^+•^ calcd. for C_22_H_26_N_2_S_6_^+•^, 510.04147; found, 510.04104; CV (vs SCE, CH_2_Cl_2_): *E*_1/2_^ox1^ = 0.46 V, *E*_1/2_^ox2^ = 0.84 V, *E*_1/2_^ox3^ = 1.06 V.

**2-[4,5-Bis(propylthio)-1,3-dithiol-2-ylidene]-5-(4-hydroxyphenyl)-5*H*-1,3-dithiolo[4,5-*c*]pyrrole (4f).** Prepared from **7a** (0.049 g, 0.125 mmol), CuI (0.012 g, 0.063 mmol), K_3_PO_4_ (0.082 g, 0.386 mmol), *trans*-diaminocyclohexane (7.5 µL, 0.062 mmol) and 4-bromophenol (**8d**, 0.035 g, 0.202 mmol) in 2 mL of dry dioxane. The product was purified by flash chromatography (CH_2_Cl_2_) to afford yellow crystals. Yield: 5 mg (0.052 mmol, 42%); Mp 123–125 °C; *R*_f_ = 0.27 (CH_2_Cl_2_); ^1^H NMR (200 MHz, CDCl_3_) δ 7.21–7.14 (m, 2H), 6.91–6.83 (m, 2H), 6.77 (s, 2H), 4.91 (s, 1H), 2.81 (t, ^3^*J* = 7.2 Hz, 4H), 1.66 (sext, ^3^*J* = 7.2 Hz, 4H), 1.01 (t, ^3^*J* = 7.2 Hz, 6H); ^13^C NMR (50 MHz, CDCl_3_) δ 153.9, 134.1, 129.7, 127.5, 122.2, 121.4, 119.2, 116.2, 111.4, 38.2, 23.1, 13.2; UV–vis (CH_2_Cl_2_) λ_max_ (ε): 309 nm (26000 L∙mol^−1^∙cm^−1^), 326 (25900); MS (EI) *m*/*z* (%): 483 (100) [M]^+•^, 440 (5) [M − Pr]^+∙^, 407 (25) [M −HSPr]^+•^; HRMS (EI) *m*/*z*: [M]^+•^ calcd. for C_20_H_20_N_2_O_2_S_6_^+•^, 482.99419; found, 482.99474; CV (vs SCE, CH_2_Cl_2_): *E*_1/2_^ox1^ = 0.47 V, *E*_1/2_^ox2^ = 0.86 V, *E*_1/2_^ox3^ = 1.77 V.

## Supporting Information

File 1 Experimental details, details on electrochemical characterization, ^1^H and ^13^C NMR spectra of compounds **4b,d–f**, UV–vis spectrum and CV of **4b**, as well as full crystal structure descriptions.

File 2 Zip archive containing X-ray crystallographic data for **4a** (CCDC 987551), **4b** (CCDC 1049639), **4d** (CCDC 1049638) and **4e** (CCDC 1049637).
