# Supplementary material for: Tuning of tetrathiafulvalene properties: versatile synthesis of N-arylated monopyrrolotetrathiafulvalenes via Ullmann-type coupling reactions
Source: Beilstein J Org Chem. 2015 May 21;11:860–8. doi: 10.3762/bjoc.11.96 (PMC4464400; doi:10.3762/bjoc.11.96)
Supplement: File 1 — Experimental details, details on electrochemical characterization, 1H and 13C NMR spectra of compounds 4b,d–f, UV–vis spectrum and CV of 4b, as well as full crystal structure descriptions. [file Beilstein_J_Org_Chem-11-860-s001.pdf]

Supporting Information

for

Tuning of tetrathiafulvalene properties: versatile synthesis of *N*-arylated monopyrrolotetrathiafulvalenes via Ullmann-type coupling reactions

Vladimir A. Azov*[1], Diana Janott[1], Dirk Schlüter[1] and Matthias Zeller[2]

Address: [1]Department of Chemistry, University of Bremen, Leobener Str. NW 2C, D-28359 Bremen, Germany and [2]One University Plaza, Department of Chemistry, Youngstown State University, Youngstown, OH 44555-3663, USA

Email: Vladimir A. Azov - vazov@uni-bremen.de

* Corresponding author

Experimental details, details on electrochemical characterization, ^{1}H and ^{13}C NMR spectra of compounds 4b,d–f, UV–vis spectrum and CV of 4b, as well as full crystal structure descriptions

Table of Content

Experimental	S2
Synthetic scheme for preparation of intermediates **7a–c**	S3
NMR spectra of compounds **4b,d–f**	S4
Additional UV–vis spectra and CV	S8
Crystal structure descriptions	S9
References	S16

Experimental

General. Monopyrrolotetrathiafulvalenes **7a**,[1] **7b**,[2] and **7c**[3] were prepared as reported previously. Reagent grade chemicals and solvents, including dry dioxane (*Acros*, extra dry, over molecular sieves) and CuI (≥99.5%), were used without further purification. All reactions were carried out under an atmosphere of dry N_2. Chemical shifts (δ) are reported in parts per million (ppm) relative to TMS, the residual solvent signals were used as reference: $CDCl_3$ (7.26 ppm for ^1H, 77.0 ppm for ^{13}C) or CD_2Cl_2 (5.32 ppm for ^1H, 53.8 ppm for ^{13}C). ^1H NMR coupling constants (J) are reported in Hertz (Hz) and multiplicity is indicated as follows: s (singlet), d (doublet), t (triplet), q (quartet), sext (sextet). High resolution MS-spectra (HRMS) were measured on a *Finnigan MAT 95* spectrometer (EI, 70 eV), UV–vis measurements were performed in a 1 cm path length quartz optical cell. Melting points were determined using a capillary melting point apparatus and are uncorrected. R_f values were determined using 0.2 mm silica gel F-254 aluminum TLC cards, which were inspected under 254 nm UV light. Flash chromatography (FC) was carried out using 230–440 mesh (particle size 36–70 μm) silica gel.

Cyclic voltammetry. Cyclic Voltammetry (CV) was performed using a computer controlled *HEKA PG390* potentiostat in a three-electrode single-compartment cell (2.5 mL) with a platinum disk working electrode (diameter of 1.5 mm) and a platinum wire used as counter electrode. A non-aqueous Ag/Ag^+ secondary electrode (0.1 M TPAP + 0.01 M $AgNO_3$ in MeCN) was used as the reference electrode. CV samples were dissolved to a concentration of 1×10^{-3} M in dry degassed CH_2Cl_2 containing 0.1 M Bu_4NClO_4 (TBAP) as supporting electrolyte. Ferrocene was used as an electrochemical reference[4] with the potential $E_{1/2}^{ox}$ = 0.48 V vs saturated calomel electrode (SCE) for the Fc/Fc^+ couple in TBAP/CH_2Cl_2.[5] Thus, a CV scan of 1×10^{-3} M ferrocene solution was taken after each CV measurement for calibration purposes. Then the values of oxidation potentials were referenced to the Fc/Fc^+ couple and recalculated and reported vs SCE.

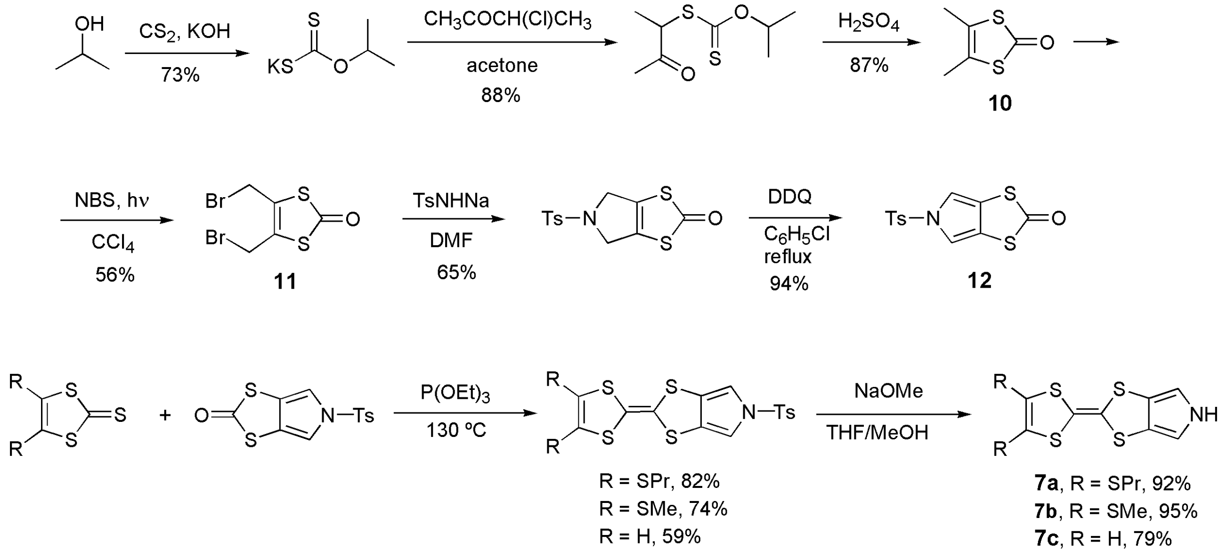

Scheme S1: Synthesis of monopyrrolo-tetrathiafulvalenes **7a–c**. 4,5-Dimethyl-1,3-dithiol-2-one **10**,[6] 4,5-bis(bromomethyl)-1,3-dithiol-2-one **11**,[7] and *N*-tosyl-1,3-dithiolo[4,5-*c*]pyrrol-2-one[8] **12** were prepared according to reported reaction procedures with some minor adjustments.

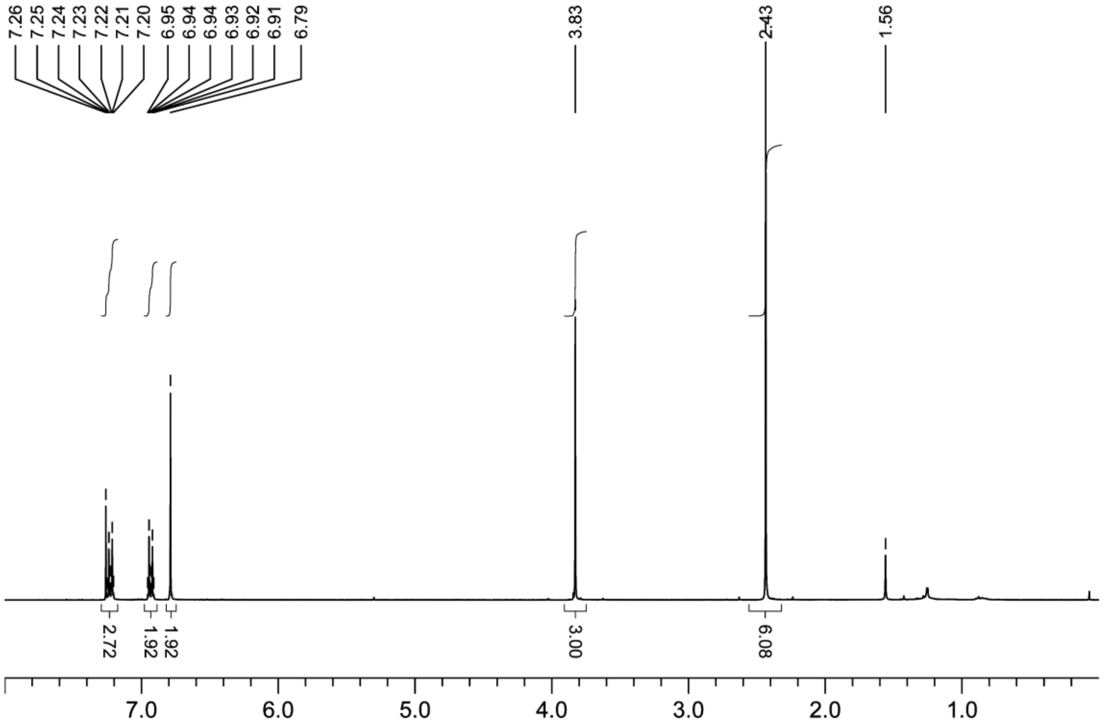

Figure S1a: ^{1}H NMR spectrum of compound **4b** (360 MHz, $CDCl_3$).

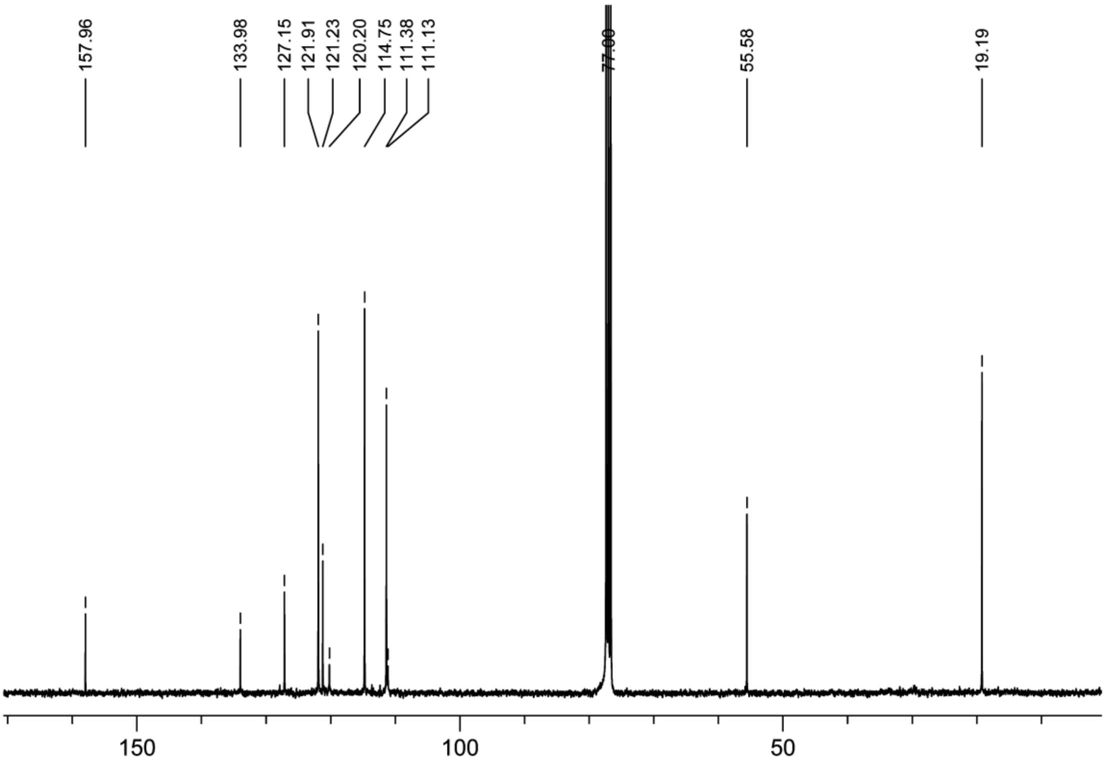

Figure S1b: ^{13}C NMR spectrum of compound **4b** (90 MHz, $CDCl_3$).

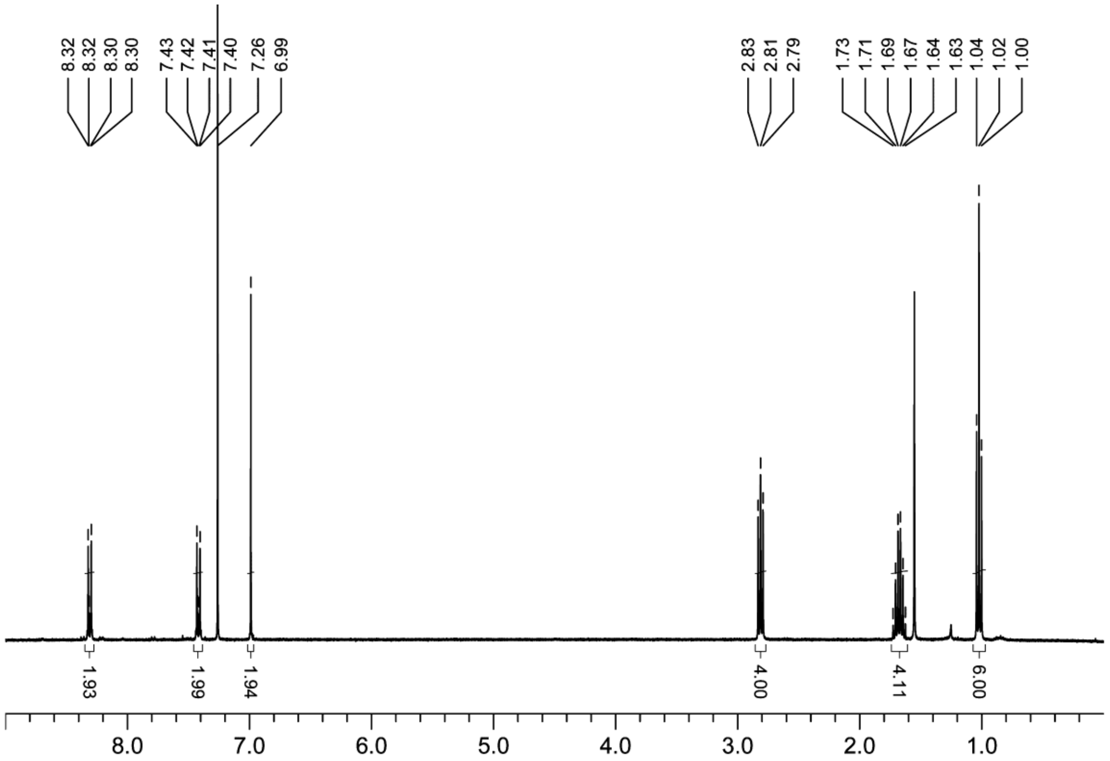

Figure S2a: ^{1}H NMR spectrum of compound **4d** (360 MHz, $CDCl_3$).

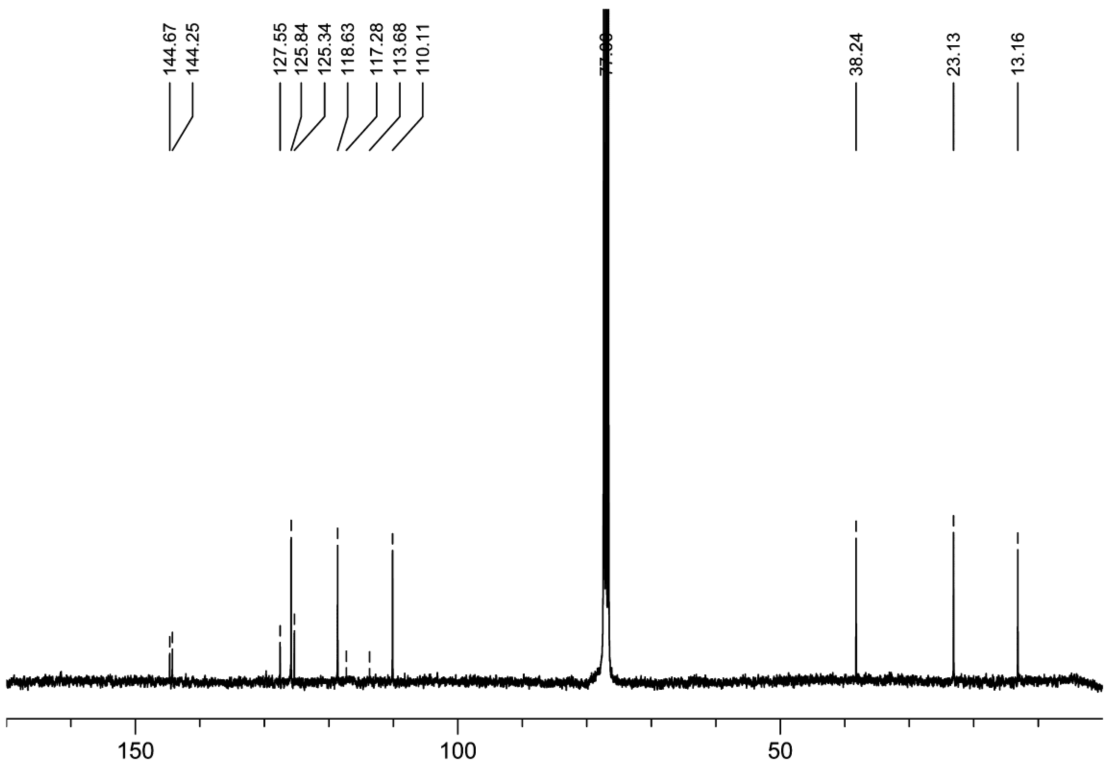

Figure S2b: ^{13}C NMR spectrum of compound **4d** (90 MHz, $CDCl_3$).

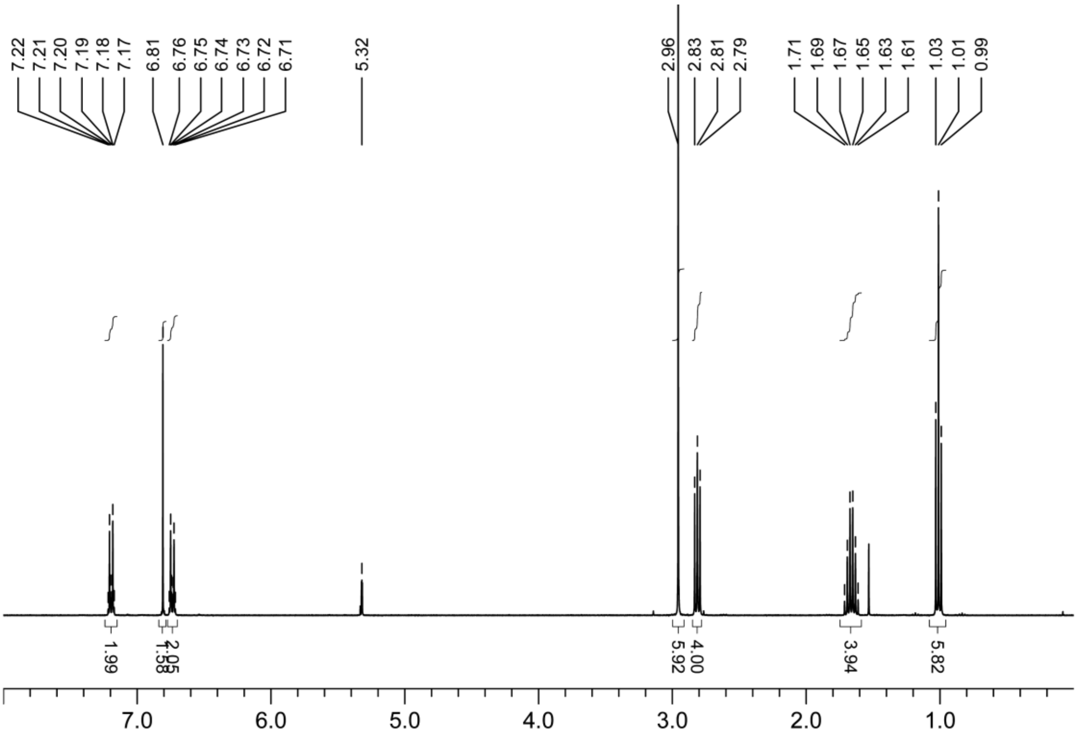

Figure S3a: ^{1}H NMR spectrum of compound **4e** (360 MHz, CD_2Cl_2).

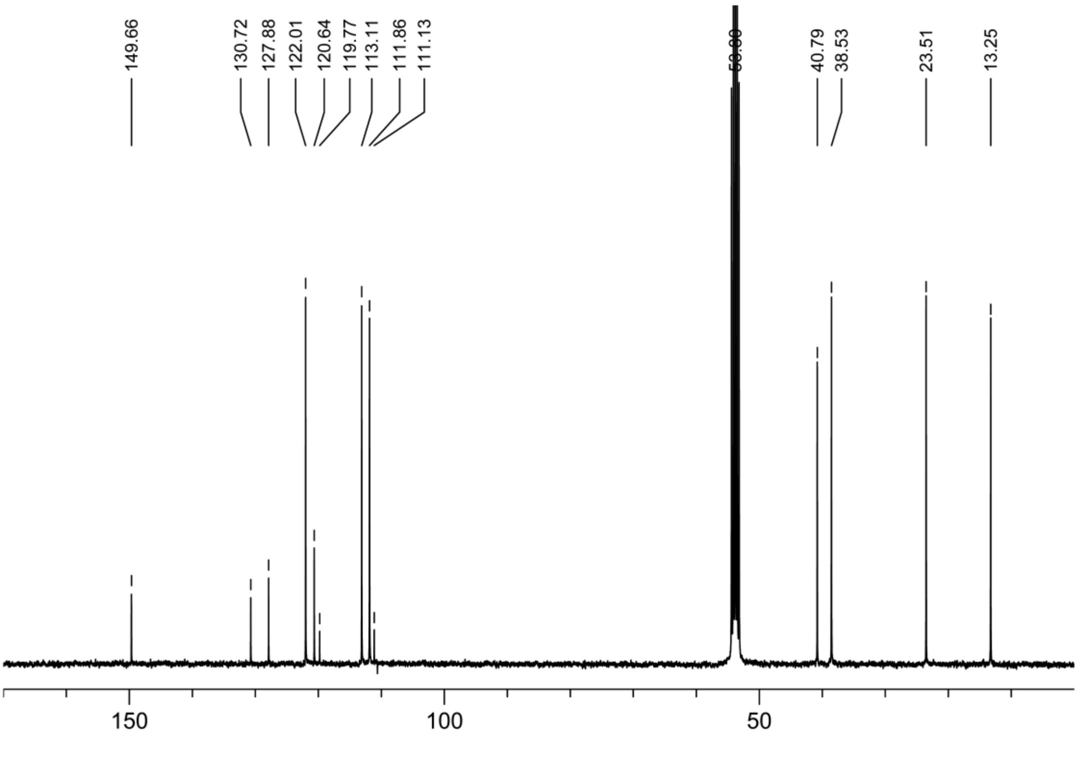

Figure S3b: ^{13}C NMR spectrum of compound **4e** (90 MHz, CD_2Cl_2).

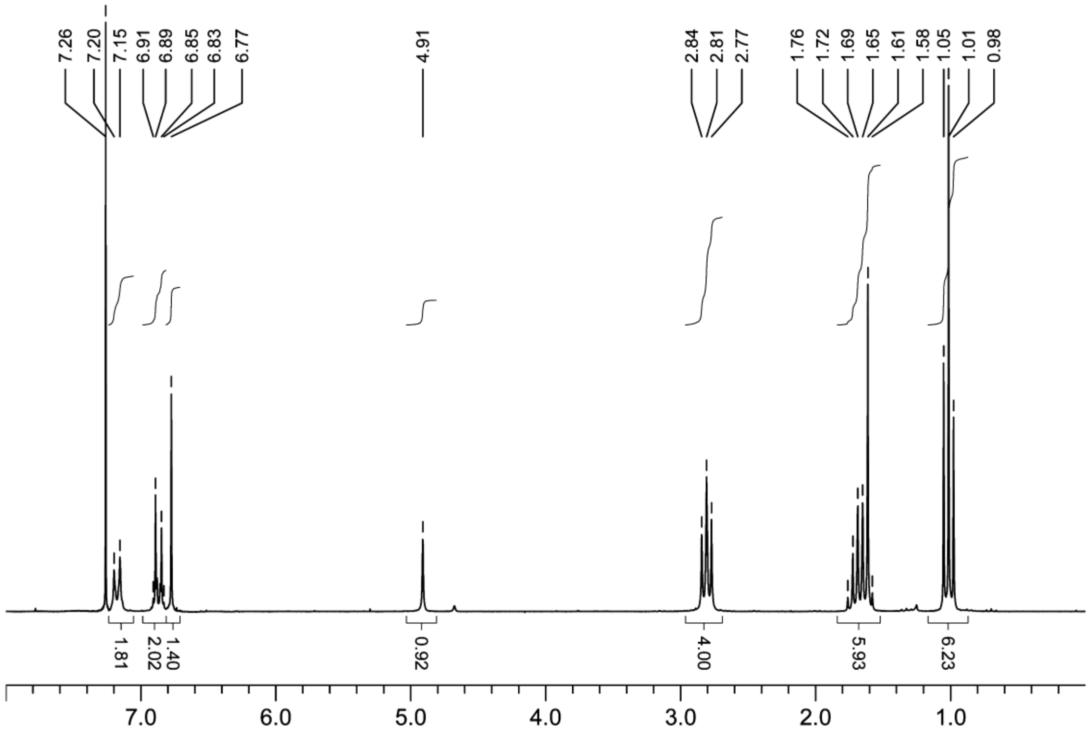

Figure S4a: ^{1}H NMR spectrum of compound **4f** (200 MHz, $CDCl_3$).

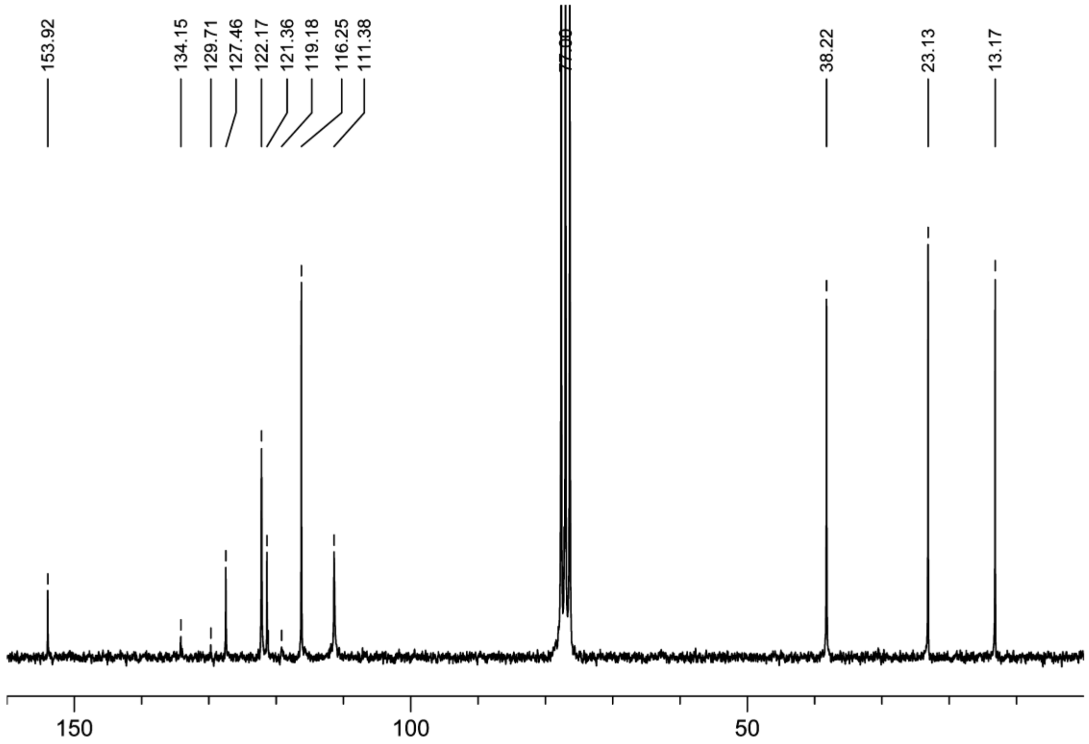

Figure S4b: ^{13}C NMR spectrum of compound **4f** (50 MHz, $CDCl_3$).

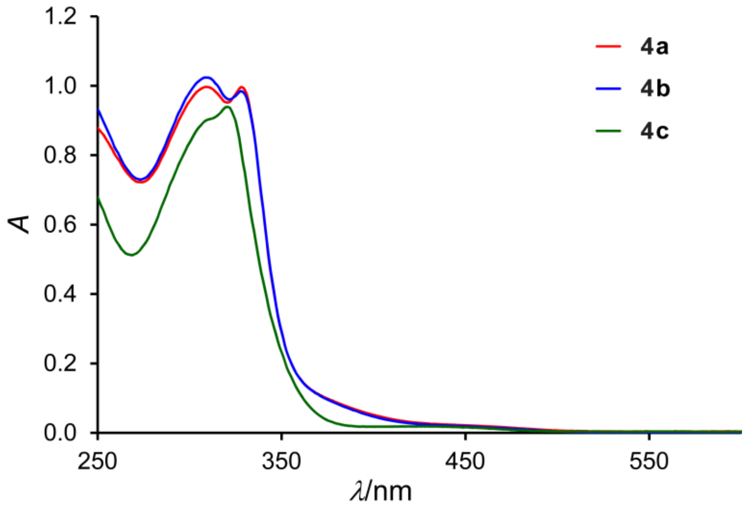

Figure S5: UV–vis spectra of compounds **4a–c**. CH_2Cl_2, $c = 4 \times 10^{-5}$ M.

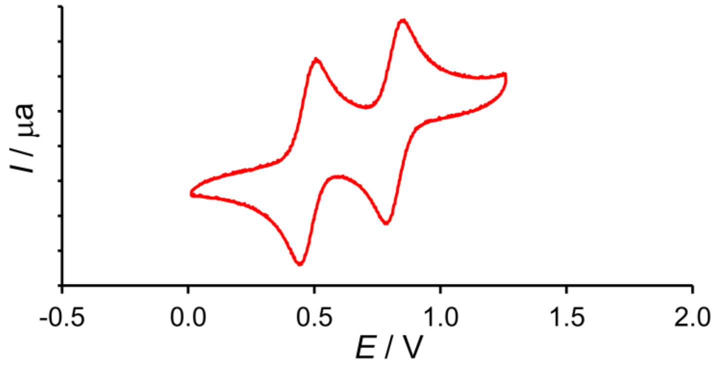

Figure S6: Cyclic voltammogram of compound **4b**. CH_2Cl_2/Bu_4NClO_4, plotted vs SCE.

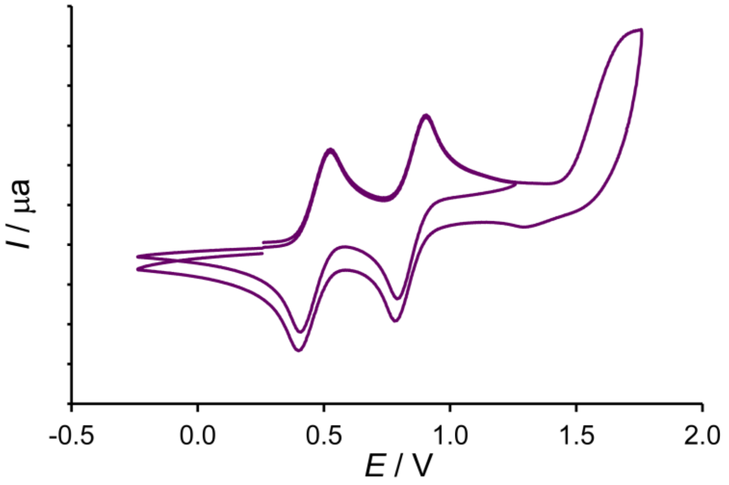

Figure S7: Cyclic voltammograms of compound **4f** recorded for narrow and wide scan windows. Additional irreversible oxidation wave is observed at higher potential. CH_2Cl_2/Bu_4NClO_4, plotted vs SCE.

X-ray crystal structure determination

Diffraction data were collected on *Bruker AXS SMART APEX* (**4a**), *Bruker AXS APEX II* (**4b**), *Bruker AXS Prospector* (**4d**) CCD diffractometers or on a *Bruker AXS D8 Quest* CMOS diffractometer (**4e**) at 100 K using monochromatic Mo Kα (**4a**, **4b**, and **4e**) or Cu Kα (**4d**) radiation with the omega and phi scan techniques. Data were collected, unit cells determined, and the data integrated and corrected for absorption and other systematic errors using the *Apex2* suite of programs.[9] The space groups were assigned and the structures were solved by direct methods using the *SHELXTL* suite of programs[10] and refined by full matrix least squares against F^2 with all reflections using *Shelxl2013*.[11] H-atoms attached to carbon atoms were positioned geometrically and constrained to ride on their parent atoms, with carbon hydrogen bond distances of 0.95, 0.99 and 0.98 Å for aromatic C–H, CH_2 and CH_3 moieties, respectively. Methyl H atoms were allowed to rotate but not to tip to best fit the experimental electron density. U_{iso}(H) values were set to a multiple of U_{eq}(C) with 1.5 for CH_3 and 1.2 for C–H and CH_2 units, respectively.

In **4a**, one of the propyl groups is disordered over two alternative positions with an occupancy ratio of 0.877(2) to 0.123(2). The minor moiety was restrained to have a similar geometry as the major one, and ADPs of equivalent atoms in both moieties were constrained to be identical. Reflection 0 0 1 was obstructed by the beam stop and was omitted from the refinement.

In the structure of **4e** two of the thiopropyl side chains show disorder. One is disordered by a pseudo-mirror operation bisecting the thiopropyl chain. The disorder of the other chain is due to close proximity to the first thiopropyl side chain, and they share a common occupancy ratio. The minor moieties were restrained to have a geometry similar to that of the major moieties, and disordered atoms were subjected to a rigid bond restraint (RIGU in Shelxl). Disordered atoms C20A and C20C were constrained to have identical ADPs. Minor twinning by pseudo-merohedry was observed. Application of the twin law 1 0 0 -1 -1 0 0 0 -1 resulted in a BASF value of 0.0083(2). Omission of twinning resulted in an increase of R value by about 0.3%. Minor whole molecule disorder is observed, with positions of sulfur atoms of the second orientation visible in difference density maps. The largest such peak accounts for 1.22 electrons, indicating a possible prevalence for the second orientation of less than 8%. In the absence of visible positions for C and N atoms, no attempt was made to resolve the disorder. Reflections 0 -1 1 and -1 0 1 were affected by the beam stop and were omitted from the refinement.

Complete crystallographic data, in CIF format, have been deposited with the Cambridge Crystallographic Data Centre. These data format can be obtained free of charge from The Cambridge Crystallographic Data Centre via www.ccdc.cam.ac.uk/data_request/cif on quoting the deposit numbers CCDC 987551 (**4a**), CCDC 1049639 (**4b**), CCDC 1049638 (**4d**), CCDC 1049637 (**4e**).

X-ray crystallographic data and refinement details for all four structures are summarized in Table S1.

Table S1: Crystal data and structure refinement for compound **4a**, **4b**, **4d**, and **4e**.

**Compound**	**4a**	**4b**	**4d**	**4e**
**Crystal data**				
Chemical formula	$C_{21}H_{23}NOS_6$	$C_{17}H_{15}NOS_6$	$C_{20}H_{20}N_2O_2S_6$	$C_{22}H_{26}N_2S_6$
M_r	497.76	441.66	512.74	510.81
Crystal system, space group	Triclinic, $P\bar{1}$	Triclinic, $P\bar{1}$	Monoclinic, $P2_1/c$	Triclinic, $P\bar{1}$
Temperature (K)	100	100	100	100
a, b, c (Å)	10.8196 (9), 12.0793 (10), 19.0960 (16)	10.761 (3), 11.927 (4), 16.090 (5)	10.9203 (13), 11.8068 (14), 34.711 (4)	11.5871 (4), 12.1431 (5), 18.8970 (8)
α, β, γ (°)	72.509 (1), 89.470 (1), 70.382 (1)	80.067 (5), 84.081 (5), 66.129 (4)	90.0, 91.759 (4), 90.0	94.0239 (14), 90.9921 (13), 117.9112 (12)
V (Å^3)	2230.5 (3)	1858.8 (10)	4473.3 (9)	2339.98 (16)
Z	4	4	8	4
Radiation type	Mo $K\alpha$	Mo $K\alpha$	Cu $K\alpha$	Mo $K\alpha$
μ (mm^{-1})	0.63	0.74	5.83	0.60
Crystal size (mm)	0.55 × 0.45 × 0.16	0.53 × 0.27 × 0.10	0.34 × 0.27 × 0.02	0.34 × 0.27 × 0.07
**Data collection**				
Diffractometer	Bruker AXS SMART APEX CCD diffractometer	Bruker AXS APEXII CCD diffractometer	Bruker AXS Prospector CCD diffractometer	Bruker AXS D8 Quest CMOS diffractometer
Absorption correction	Multi-scan, SADABS, Bruker Apex2	Multi-scan, SADABS, Bruker Apex2	Multi-scan, SADABS, Bruker Apex2	Multi-scan, SADABS, Bruker Apex2
T_{min}, T_{max}	0.675, 0.746	0.588, 0.746	0.503, 0.753	0.619, 0.746
No. of measured, independent and observed [$I > 2\sigma(I)$] reflections	51562, 14172, 12316	18740, 10894, 9224	32114, 7651, 7112	52004, 14183, 11758
R_{int}	0.018	0.026	0.048	0.050
$(\sin \theta/\lambda)_{max}$ (Å^{-1})	0.748	0.734	0.598	0.715
**Refinement**				
$R[F^2 > 2\sigma(F^2)]$, $wR(F^2)$, S	0.027, 0.070, 1.03	0.038, 0.108, 1.02	0.085, 0.213, 1.22	0.048, 0.116, 1.08
No. of reflections	14172	10894	7651	14183
No. of parameters	540	457	545	610
No. of restraints	3	-	-	72
H-atom treatment	H-atom parameters constrained	H-atom parameters constrained	H-atom parameters constrained	H-atom parameters constrained
$\Delta\rho_{max}$, $\Delta\rho_{min}$ (e Å^{-3})	0.50, -0.29	0.81, -0.40	1.04, -1.14	1.22, -0.59

Table S2a: Parameters of selected weak intermolecular hydrogen-bonds for **4a**.

D—H···*A*	*D*—H (Å)	H···*A* (Å)	*D*···*A* (Å)	*D*—H···*A* (°)
C15A—H15B···S5B^{i}	0.98	2.94	3.5690 (13)	122.9
C16A—H16A···O1A^{ii}	0.99	2.58	3.2905 (15)	128.4
C16A—H16B···S3B^{iii}	0.99	2.93	3.7666 (13)	143.4

Symmetry code(s): (i) −*x*, −*y*+1, −*z*; (ii) −*x*, −*y*, −*z*; (iii) *x*, *y*−1, *z*.

Table S2b: Parameters of short intermolecular contacts involving S-atoms for **4a**.

S···*A*	*S*···*A* (Å)
S6A···S4B^{iv}	3.4115(6)
S1B···S1B^{v}	3.4457(4)
S5A···C9B^{iii}	3.3819(12)
S6B···N1A^{vi}	3.3117(11)

Symmetry code(s): (iii) *x*, *y*−1, *z*; (iv) *x* −1, *y*−1+, *z*; (v) *x*−2, -*y*, *z*−1; (vi) −*x*+1, −*y*+1, −*z*.

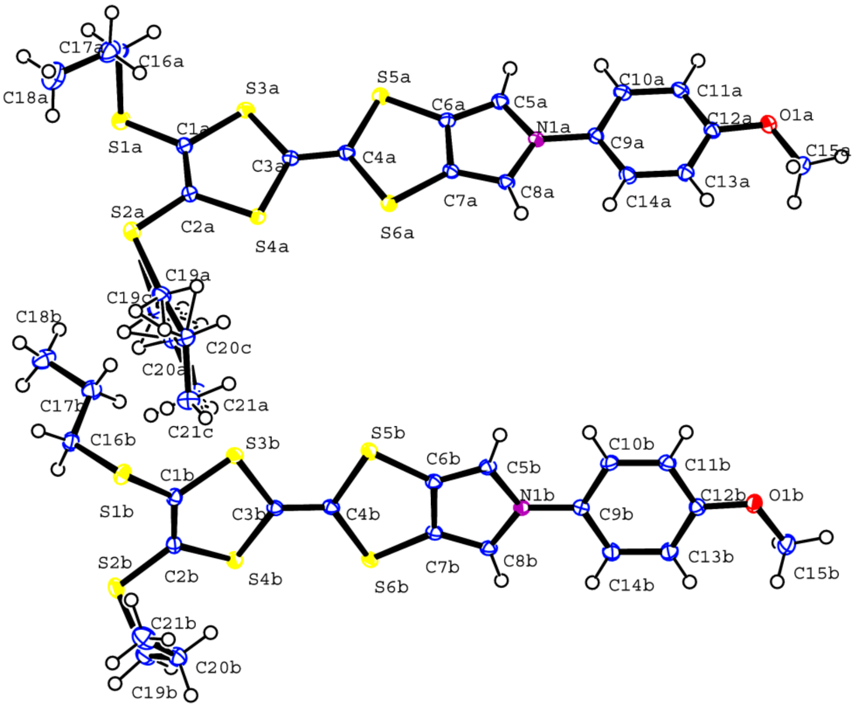

Figure S8: ORTEP plot of **4a** showing the two independent molecules. One of the propyl chains is disordered between two positions in an 87.7 (C19a-C20a-C20a) to 12.3 (C19c-C20c-C20c) ratio. Thermal ellipsoids are shown at the 50% probability level.

Table S3a: Parameters of selected weak intermolecular hydrogen-bonds for **4b**.

D—H···*A*	*D*—H (Å)	H···*A* (Å)	*D*···*A* (Å)	*D*—H···*A* (°)
C4B—H4B···S1A^{i}	0.95	2.99	3.9319 (18)	169.9
C10A—H10A···S1B^{ii}	0.95	3.00	3.8880 (18)	156.2
C11B—H11B···S3A^{i}	0.95	2.92	3.7093 (19)	141.9
C15A—H15B···S2B^{iii}	0.98	3.02	3.5394 (19)	114.7
C15A—H15C···S6A^{iv}	0.98	2.88	3.686 (2)	140.0
C16B—H16A···O1A^{i}	0.98	2.61	3.282 (2)	125.8
C16B—H16B···S5A^{v}	0.98	2.88	3.792 (2)	154.6
C18B—H18A···O1A^{iii}	0.98	2.53	3.128 (2)	119.6

Symmetry code(s): (i) −*x*+1, −*y*+1, −*z*; (ii) *x*−1, *y*+1, *z*; (iii) −*x*, −*y*+1, −*z*; (iv) *x*−1, *y*+1, *z*−1; (v) −*x*+2, −*y*, −*z*+1.

Table S3b: Parameters of short intermolecular contacts involving S-atoms for **4b**.

S···*A*	*S*···*A* (Å)
S1A ··S1B	3.2570(18)
S2A···S3B^{ii}	3.5729(14)
S2A···C7B^{ii}	3.461(2)
S6B···S6B^{vi}	3.5676(14)

Symmetry code(s): (ii) *x*−1, *y*+1, *z*; (vi) −*x*+2, −*y*−1, −*z*+1.

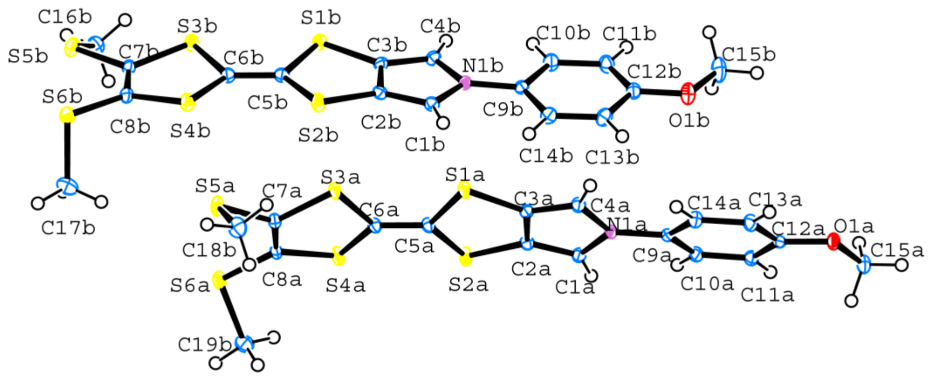

Figure S9: ORTEP plot of **4b** showing the two independent molecules. Thermal ellipsoids are shown at the 50% probability level.

Table S4a: Parameters of selected weak intermolecular hydrogen-bonds for **4d**.

D—H···*A*	*D*—H (Å)	H···*A* (Å)	*D*···*A* (Å)	*D*—H···*A* (°)
C18—H18A···O3^{i}	0.99	2.53	3.427 (9)	150.8
C19—H19A···O4^{i}	0.99	2.63	3.374 (9)	131.7
C30—H30···S9^{ii}	0.95	3.02	3.861 (7)	147.7
C31—H31···S7^{ii}	0.95	2.98	3.896 (7)	163.3
C38—H38A···O3^{ii}	0.99	2.42	3.354 (10)	156.7
C38—H38B···O1^{iii}	0.99	2.64	3.450 (9)	138.6

Symmetry code(s): (i) −*x*+2, *y*−1/2, −*z*+1/2; (ii) −*x*+1, *y*+1/2, −*z*+1/2; (iii) −*x*+2, *y*+1/2, −*z*+1/2.

Table S4b: Parameters of short intermolecular contacts involving S-atoms for **4d**.

S···*A*	*S*···*A* (Å)
S4···S11	3.524(2)
S11···C2	3.466(7)
S4···S12^{iv}	3.513(2)
S6···S10^{iv}	3.567(2)
S6···S12^{iv}	3.574(2)

Symmetry code(s): (iv) −*x*+1, *y*−1/2, −*z*+1/2.

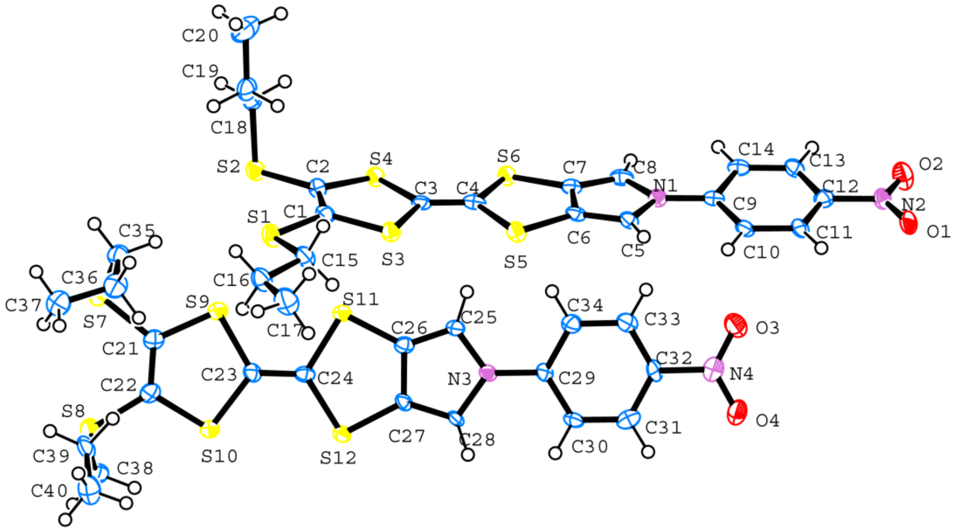

Figure S10: ORTEP plot of **4d** showing the two independent molecules. Thermal ellipsoids are shown at the 50% probability level.

Table S5a: Parameters of selected weak intermolecular hydrogen-bonds for **4e**.

D—H···*A*	*D*—H (Å)	H···*A* (Å)	*D*···*A* (Å)	*D*—H···*A* (°)
C5A—H5A···N2B[i]	0.95	2.53	3.314 (3)	139.4
C8A—H8A···S6B[ii]	0.95	2.94	3.884 (2)	175.5
C17A—H17A···S4B[iii]	0.99	2.96	3.857 (4)	151.0
C17A—H17B···S2B[iv]	0.99	2.74	3.697 (4)	161.9
C21A—H21B···S1B	0.99	2.96	3.913 (14)	161.3
C21C—H21F···S1B	0.99	3.02	3.865 (17)	144.6
C5B—H5B···S5A[v]	0.95	2.96	3.908 (2)	172.9
C18B—H18D···S1C[iv]	0.99	2.90	3.694 (8)	137.7

Symmetry code(s): (i) −*x*+1, −*y*+2, −*z*+1; (ii) −*x*, −*y*+1, −*z*+1; (iii) *x*, *y*−1, *z*; (iv) −*x*, −*y*, −*z*; (v) *x*, *y*+1, *z*.

Table S5b: Parameters of short intermolecular contacts involving S-atoms for **4e**.

S···*A*	*S*···*A* (Å)
S6B···N1A	3.2548(19)
S4A···S5B[iii]	3.5176(9)
S5A···N1B[ii]	3.3375(19)
S5A···C9B[ii]	3.482(2)

Symmetry code(s): (iii) *x*, *y*−1, *z*; (ii) −*x*, −*y*+1, −*z*+1.

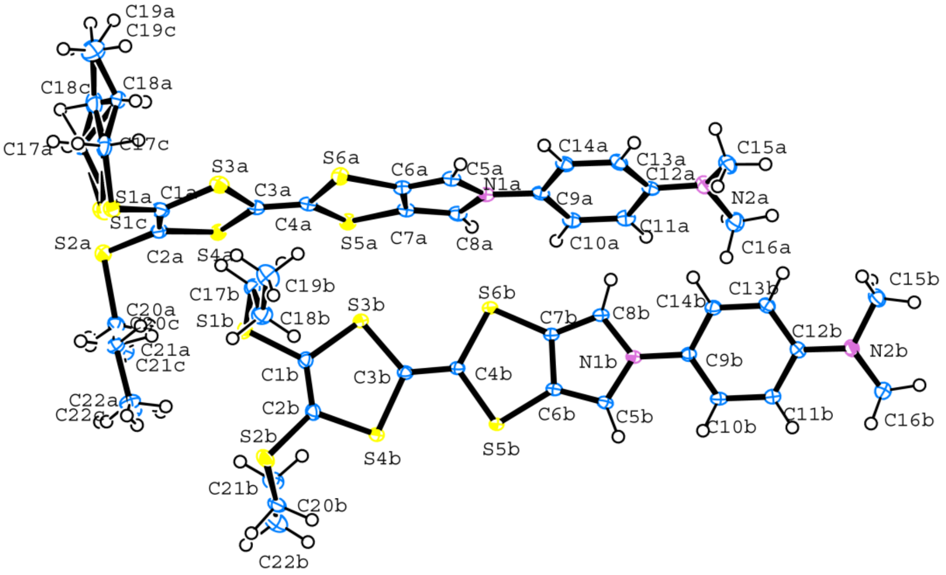

Figure S11: ORTEP plot of **4e** showing the two independent molecules. Two thiopropyl chains are disordered between two positions in a 58.7 (S1a-C17a-C18a-C19a and C20a-C21a-C22a) to 41.3 (S1c-C17c-C18c-C19c and C20c-C21c-C22c) ratio. Thermal ellipsoids are shown at the 50% probability level.

References

1. Hansen, J. A.; Becher, J.; Jeppesen, J. O.; Levillain, E.; Nielsen, M. B.; Petersen, B. M.; Petersen J. C.; Sahin, Y. *J. Mater. Chem.* **2004**, *14*, 179–184.
2. Jeppesen, J. O.; Takimiya, K.; Jensen, F.; Brimert, T.; Nielsen, K.; Thorup, N.; Becher, J. *J. Org. Chem.* **2000**, *65*, 5794–5805.
3. Nygaard, S.; Hansen, C. N.; Jeppesen, J. O. *J. Org. Chem.* **2007**, *72*, 1617–162.
4. a) Gritzner, G.; Kuta, J. *Pure Appl. Chem.* **1984**, *56*, 461–466; b) Pavlishchuk, V. V.; Addison, A. W. *Inorg. Chim. Acta* **2000**, *298*, 97–102; c) Connely, N. G.; Geiger, W. E. *Chem. Rev.* **1996**, *96*, 877–910.
5. a) Chang, D.; Malinski, T.; Ulman, A.; Kadish, K. M. *Inorg. Chem.* **1984**, 23, 817–824; b) Rosokha, S. V.; Kochi, J. K. *J. Am. Chem. Soc.* **2007**, *129*, 3683–3697
6. Chandrasekaran, P.; Donahue, J. P. *Org. Synth.* **2009**, *86*, 333–343.
7. Crivillers, N.; Oxtoby, N. S.; Mas-Torrent, M.; Verciana, J.; Rovira, C. *Synthesis* **2007**, *11*, 1621–1623.
8. Jeppesen, J. O.; Takimiya, K.; Jensen, F.; Brimert, T.; Nielsen, K.; Thorup, N.; Becher, J. *J. Org. Chem.* **2000**, *65*, 5794–5805.
9. *Apex2,* Bruker Advanced X-ray Solutions, Bruker AXS Inc., Madison, Wisconsin, USA, 2013.
10. a) *SHELXTL* (Version 6.14) Bruker Advanced X-ray Solutions, Bruker AXS Inc., Madison, Wisconsin, USA, 2000-2003. b) Sheldrick, G. M. *Acta Cryst.* **2008**, *A64*, 112-122.
11. Sheldrick, G. M. University of Göttingen, Germany, 2013-2014.
